# Regulation of LRRK2 Stability by the E3 Ubiquitin Ligase CHIP

**DOI:** 10.1371/journal.pone.0005949

**Published:** 2009-06-17

**Authors:** Xiaodong Ding, Matthew S. Goldberg

**Affiliations:** 1 Department of Neurology, The University of Texas Southwestern Medical Center, Dallas, Texas, United States of America; 2 Department of Psychiatry, The University of Texas Southwestern Medical Center, Dallas, Texas, United States of America; National Institutes of Health, United States of America

## Abstract

Dominantly inherited mutations in the leucine-rich repeat kinase 2 gene (LRRK2) are the most common cause of familial Parkinson's disease (PD) and have also been identified in individuals with sporadic PD. Although the exact cellular function of LRRK2 remains unknown, most PD-linked mutations appear to be toxic to cells in culture via mechanisms that depend on the kinase activity of LRRK2 or on the formation of cytoplasmic inclusions. Here we show that the E3 ubiquitin ligase CHIP physically associates with LRRK2 and regulates the cellular abundance of LRRK2. We further show that LRRK2 forms a complex with overexpressed and endogenous CHIP and Hsp90. Our data indicates that the destabilization of LRRK2 by CHIP is due to ubiquitination and proteasome-dependent degradation. Hsp90 can attenuate CHIP-mediated degradation and this can be blocked by the Hsp90 inhibitor geldanamycin. These findings provide important insight into the cellular regulation of LRRK2 stability and may lead to the development of therapeutics to treat PD based on controlling LRRK2 stability.

## Introduction

Parkinson's disease (PD) is the most common neurodegenerative movement disorder and affects 1–2% of the population over 60 years old. The primary clinical symptoms have classically been defined as bradykinesia, resting tremor, cogwheel rigidity and abnormal gait. Despite intensive research, the cause of PD remains unknown. Epidemiological studies have failed to definitively identify a single cause for PD, however, age is the greatest risk factor and many studies have implicated cumulative oxidative stress, mitochondrial dysfunction, protein aggregation and neuroinflammation in PD pathogenesis or progression [1], [2], [3], [4]. Although PD is generally sporadic, many families are known to have a Mendelian pattern of inherited parkinsonism. The recent identification of several genes with mutations linked to familial forms of PD has provided unprecedented opportunities to discover potential pathogenic mechanisms and to rationally develop more effective therapies [5].

Of all the mutations linked to familial PD to date, the G2019S missense mutation in the leucine-rich repeat kinase 2 gene (LRRK2) is the most common and can account for a significant fraction of apparently sporadic PD cases in certain populations [6], [7], [8]. LRRK2 is expressed widely throughout the brain and other tissues, including—but not limited to—the cells most affected in PD [9], [10], [11]. The LRRK2 gene codes for a large protein of 2527 amino acids with multiple domains predicted by primary sequence homology [12]. The N-terminus is predicted to contain armadillo (ARM) repeats and ankyrin repeats, followed by a leucine-rich repeat (LRR) domain, a Ras of complex (Roc) GTPase domain, a C-terminal of Roc (COR) domain, a tyrosine kinase domain with greatest sequence similarity to the mixed-lineage kinase subclass of mitogen-activated protein kinase kinase kinases (MAPKKKs), followed by a C-terminal WD40 domain. The recent crystal structure of the ROC domain reveals a dimeric GTPase structure [13]. *In vitro* studies have confirmed that LRRK2 possesses both GTPase and kinase activities, including autophosphorylation activity and phosphorylation activity towards generic substrates and potential physiologic substrates [14], [15], [16], [17], [18], [19], [20], [21], [22].

Dominantly inherited point mutations that segregate with PD have been identified in the leucine-rich repeat, ROC GTPase, COR and kinase domains of LRRK2 [12]. Additional putatively pathogenic amino acid substitutions have been identified in these domains and in the ARM and ankyrin repeat regions and the WD40 domain of LRRK2 [23]. The molecular mechanisms by which these mutations cause PD remain uncertain. The prevailing hypothesis is that these mutations either directly or indirectly lead to increased LRRK2 kinase activity and promote inclusion formation, which can be neurotoxic [24]. Because point mutations have been identified throughout the sequence of LRRK2, not just in the kinase domain, it is likely that intra- and intermolecular protein-protein interactions both inside and outside the kinase domain are important for the normal function of LRRK2. Alterations in protein-protein interactions may be the mechanism by which some LRRK2 mutations cause disease. Identifying proteins that interact with LRRK2 is therefore crucial for filling the current gap in our understanding of the normal function of LRRK2 and for determining how disease-linked mutations lead to aberrant LRRK2 function and neurodegeneration.

In an effort to better define the cellular function of LRRK2, we conducted yeast two-hybrid screens to identify potential LRRK2 interacting proteins. We identified a robust protein-protein interaction between LRRK2 and CHIP (C-terminus of Hsp70-Interacting Protein), which is an E3 ubiquitin ligase crucial for the ubiquitination of several heat shock protein (HSP)70/90 client proteins that are involved in neurodegenerative disease [25]. Our screen also identified a robust protein-protein interaction between LRRK2 and Hsp90, which has previously been identified as a LRRK2 binding protein [18], [26], [27]. We confirmed both these interactions by different reporter assays in yeast cells and by co-immunoprecipation assays in mammalian cells. We further found that LRRK2 is destabilized by CHIP. The destabilization of LRRK2 by CHIP is due to CHIP-mediated ubiquitination and proteasome-dependent degradation. Hsp90 can block CHIP-mediated degradation and inhibition of Hsp90 restores CHIP-mediated degradation of LRRK2. These findings reveal potential cellular mechanisms that regulate LRRK2 abundance, which may provide therapeutic targets for familial and sporadic PD.

## Materials and Methods

### Plasmid Constructs

Full-length mouse LRRK2 cDNA was cloned by PCR from mouse brain cDNA prepared in our lab. Seven putative LRRK2 domains comprising amino acids 1–690 (ARM), 691–1009 (Ankyrin), 1010–1312 (LRR), 1331–1512 (ROC), 1514–1866 (COR), 1869–2128 (kinase) and 2141–2432 (WD40) were subcloned into pGBKT7 (Clontech) as baits for yeast two-hybrid analyses and into pMyc-CMV (Clontech) for mammalian cell transfections, along with full-length LRRK2. Point mutations (G2019S, R1441C and D1994A) were generated using the QuickChange Site-Directed Mutagenesis kit (Stratagene). Wild-type human LRRK2 and LRRK2(R1441C) constructs [28] were kindly provided by Dr. Mark Cookson. pcDNA-CHIP was kindly provided by Dr. Cam Patterson. pMT-HA-Ub was kindly provided by Dr. Pat Gallagher. pcDNA-His6-CHIP(K30A) and pcDNA-His6-CHIP(H260Q) were kindly provided by Dr. Len Neckers. pFlag-Hsp90 was kindly provided by Dr. Kapil Bhalla. pCS2-UbK0 was kindly provided by Dr. Ken Nephew. pHA-CHIP(K30A) and pHA-CHIP(H260Q) were constructed using pcDNA-His6-CHIP(K30A) and pcDNA-His6-CHIP(H260Q). pHA-CHIP, pHA-CHIPΔT, pHA-CHIPΔU, pHA-TPR and pHA-TPR(K30A) were constructed in this study by PCR cloning from pcDNA-CHIP or pcDNA-His6-CHIP(K30A).

### Antibodies and Other Reagents

The following antibodies and other reagents were used in these studies: Mouse monoclonal anti-HA (#MMS-101P Covance, Emeryville, CA); Mouse monoclonal anti-myc (#GTX20032 GeneTex, San Antonio, TX); Rabbit anti-HA (#ab9110 Abcam, Cambridge, MA); Rabbit anti-myc (#2272 Cell Signaling Technology, Danvers, MA); Rat monoclonal anti-Hsp90 (Stressgen, Ann Arbor, MI); Mouse anti-β-actin (#AAN02 Cytoskeleton, Denver, CO); Rabbit anti-CHIP (#PC711 Calbiochem, San Diego, CA); Mouse anti-Flag (#F3165 Sigma). Mouse anti-ubiquitin was kindly provided by Dr. Amyn Habib. Rabbit IgG, Peroxidase-conjugated goat anti-mouse IgG(H+L) and anti-rabbit IgG(H+L) and anti-rat IgG(H+L) were purchased from Jackson ImmunoResearch Laboratories. Lipofectamine 2000 (11668–027) was purchased from Invitrogen (Carlsbad, CA) and complete protease inhibitor cocktail (11873580001) was purchased from Roche Molecular Biochemicals (Indianapolis, IN). Geldanamycin was purchased from Alexis Biochemicals (San Diego, CA). Lactacystin (70980) was purchased from Cayman Chemical (Ann Arbor, MI). ImmunoPure Immobilized Protein A/G beads (20421) were purchased from Pierce (Rockford, IL). X-α-Gal (MESP-1900) was purchased from Growcells (Irvine, CA). X-β-Gal (X1001-5) was purchased from Zymo Research (Orange, CA). Yeast nitrogen base w/o amino acids (291940) and amino acid Drop-out supplements (DO-Leu, DO-Trp, DO-Leu-Trp, DO-Leu-Trp-His, DO-Leu-Trp-His-Ade) were from Clontech. 3-AT (3-amino-1,2,4-triazole) was purchased from Sigma. ON-TARGET*plus* SMARTpool CHIP siRNA (L-007201-00) and non-targeting control siRNA (D-001810-02-05) were purchased from Dharmacon (Lafayette, CO).

### Yeast Two-Hybrid Analyses

Bait plasmids consisting of each individual LRRK2 domain in pGBKT7 (Clontech) were transformed into yeast strain AH109 (*MATa, trp1-901, leu2-3, 112, ura3-52, his3-200, gal4*Δ, *gal80*Δ, *LYS2::GAL1_UAS_-GAL1_TATA_-HIS3, GAL2_UAS_-GAL2_TATA_-ADE2, URA3::MEL1_UAS_-MEL1_TATA_-lacZ*) (Clontech). Yeast clones harboring the bait plasmids were selected on SD/-Trp medium. Bait autoactivation was tested on SD/-Trp-His medium. The baits without or with slight autoactivation were used to screen a mouse brain cDNA library (Clontech catalog number 638863) pre-transformed in yeast strain Y187 (*MAT*α, *ura3-52, his3-200, ade2-101, trp1-901, leu2–3, 112, gal4Δ, met–, gal80Δ, URA3::GAL1UAS-GAL1TATA-lacZ*) (Clontech). Yeast two-hybrid screens were performed as previously described [29] using the Matchmaker GAL4 system (Clontech). Briefly, AH109 yeast cells harboring one bait plasmid were mated with Y187 cells harboring the Clontech Matchmaker mouse brain cDNA library. The diploid zygotes (5×10^6^) were plated on triple selective medium (SD/-Leu-Trp-His plus 5 mM 3-AT). The prey plasmids from yeast colonies growing on the triple selective medium were rescued and sequenced. To further verify the interactions in yeast cells, the obtained prey plamsids and their bait plasmid were co-transformed into AH109 yeast cells. After growing up on double selective medium (SD/-Trp-Leu), the yeast cells were re-streaked onto quadruple selective medium (SD/-Leu-Trp-His-Ade plus 2 mM 3-AT) and were used to perform X-α-Gal assays (*MEL1* reporter) and X-β-Gal colony-lift assays (*Lac*Z reporter) according to the Yeast Protocols Handbook (Clontech).

### Cell Transfection

HEK293 cells were maintained in DMEM with 4 mM L-glutamine, 4.5 g/liter glucose, 10% FBS, 50 U penicillin, 50 µg/ml streptomycin. Neuroblastoma SH-SY5Y cells were maintained in DMEM/F12 (1∶1) medium with 10% FBS and 50 U penicillin, 50 µg/ml streptomycin. For HEK293 cell transfection, cells (80% confluence) were transfected with an equal amount of total plasmid DNA (adjusted with the corresponding empty vector) by the calcium phosphate method. For siRNA transfection into SH-SY5Y cells, the cells (40–50% confluence) were transfected with 50 ng of pMyc-LRRK2 and 0–40 picomoles of CHIP siRNA or control siRNA using Lipofectamine 2000 cell transfection reagent according to the manufacturer's instructions.

### Immunoprecipitation and Western blot

Twenty-four to sixty hours after cell transfection, the cells were lysed in 500 µl ice-cold buffer (50 mM Tris pH 7.4, 150 mM NaCl, 1% NP-40, 1× Roche complete protease inhibitor cocktail). The cell lysates were incubated on ice for 30 min and centrifuged at 12,000g for 10 min at 4°C. The supernatants were pre-cleared at 4°C for 1 h with 10 µl of ImmunoPure Immobilized Protein A/G beads. Rabbit polyclonal antibody (anti-myc or anti-HA) was added to the pre-cleared supernatants at 1∶500 dilution and incubated at 4°C for 2 h. 20 µl of ImmunoPure Immobilized Protein A/G beads was added and incubated for another 1 h. The beads were washed five times in ice-cold lysis buffer with 1× protease inhibitor cocktail. Proteins were eluted from the beads by heating at 95°C in 1× Laemmli buffer, resolved on 4–20% gradient gels (BioRad), transferred to nitrocellulose and detected by western blot using mouse monoclonal anti-HA or anti-myc antibodies, HRP-conjugated goat anti-mouse secondary antibodies (Jackson Immunoresearch) and chemiluminescent detection (Pierce).

### Ubiquitination Assay

pMyc-LRRK2, pcDNA-CHIP and pMT-HA-Ub were co-transfected into HEK293 cells. After incubation for 24 h, immunoprecipitation was performed using a rabbit polyclonal anti-myc antibody. Mouse monoclonal anti-ubiquitin antibody was used to detect the ubiquitination of LRRK2. Monoclonal anti-myc antibody was used to probe LRRK2 expression.

## Results

### Identification of Hsp90 and CHIP as LRRK2 interacting proteins

**Figure 1:**
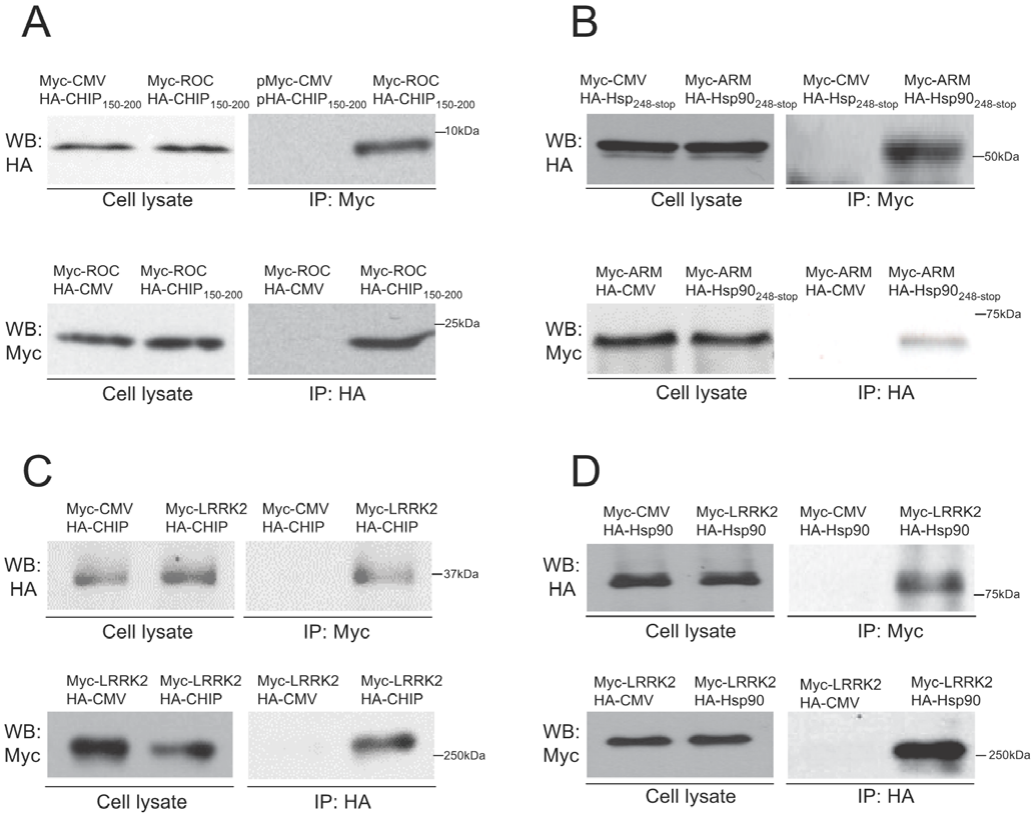
Co-immunoprecipitation of partial and full-length LRRK2, CHIP and Hsp90. The indicated myc- and HA-epitope tagged constructs (0.5 µg for each) were co-transfected into 4.5×10^6^ HEK293 cells in 60 mm dishes. Lactacystin was added to the medium at a final concentration of 5 µM to prevent CHIP-mediated degradation of LRRK2. HEK293 cell lysates were subjected to immunoprecipitation using polyclonal anti-myc or anti-HA antibodies, then immunoblotted using monoclonal anti-HA or anti-myc antibodies, respectively. Cell lysates were also directly immunoblotted using monoclonal anti-HA and anti-myc antibodies to verify similar protein expression levels in experimental and empty expression vector (CMV) control transfections. (A) The ROC domain of LRRK2 co-immunoprecipitates with its interactor identified from the yeast two-hybrid screen, CHIP_150–200_. (B) The ARM domain of LRRK2 co-immunoprecipitates with its interactor identified from the yeast two-hybrid screen, Hsp90_248–stop_. (C) Full-length LRRK2 co-immunoprecipitates with full-length CHIP. (D) Full-length LRRK2 co-immunoprecipitates with full-length Hsp90.I

We used each individual domain of LRRK2 as baits for yeast two-hybrid analyses to identify potential interacting proteins. Our screens of a mouse brain cDNA library yielded clones showing robust protein-protein interactions in yeast cells with the ARM domain and the ROC domain of LRRK2. DNA sequencing revealed that the interactor of the ARM domain corresponded to the C-terminal portion of heat shock protein 90 (Hsp90) from amino acid 248 to amino acid 704 (designated here as Hsp90_248-stop_) and the interactor of the ROC domain corresponded to the charged domain of the E3 ubiquitin ligase CHIP from amino acid 150 to amino acid 200 (designated here as CHIP_150–200_). To confirm these protein-protein interactions in mammalian cells, the LRRK2 ARM and ROC domains and their interactors (Hsp90_248–stop_ and CHIP_150–200_) were cloned into mammalian cell expression vectors with N-terminal myc or HA epitope tags (pMyc-CMV and pHA-CMV). Full-length LRRK2 and CHIP were also cloned into these vectors for mammalian cell expression. Co-transfection of HEK293 cells followed by immunoprecipitation and western blotting showed that the ROC domain of LRRK2 robustly co-immunoprecipated with CHIP_150–200_ (Figure 1A, right lane), and the ARM domain of LRRK2 co-immunoprecipitated with Hsp90_248–stop_ (Figure 1B, right lane). Full-length LRRK2 also co-immunoprecipitated with full-length CHIP and full-length Hsp90 (Figure 1C, D, right lane of each panel). All of these interactions were detected both by immunoprecipitating myc-tagged LRRK2 (or myc-LRRK2 domain) and western blotting HA-tagged interactors and by immunoprecipitating HA-tagged interactors and western blotting myc-tagged LRRK2 (or myc-LRRK2 domain). Control immunoprecipitations from cells transfected with equivalent amounts of empty CMV expression vectors (pMyc-CMV or pHA-CMV) confirmed the absence of non-specific binding to the immunoprecipitation antibodies or protein A/G beads. Direct western blotting of the cell lysates indicated that the immunoprecipitation results were not due to differences in protein expression levels or transfection efficiencies (Figure 1A–D, left two lanes of each panel).

### CHIP interacts with LRRK2 through its TPR and charged domains

**Figure 2:**
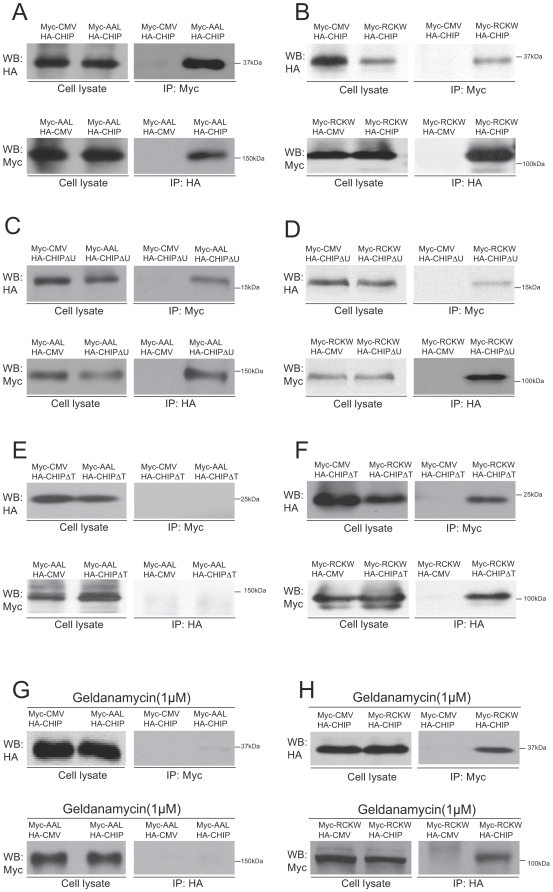
CHIP binds to multiple domains of LRRK2 by different mechanisms. The indicated constructs (0.5 µg for each) were co-transfected into 4.5×10^6^ HEK293 cells in 60 mm dishes. Lactacystin was added to the medium at a final concentration of 5 µM to prevent CHIP-mediated degradation of LRRK2. HEK293 cell lysates were subjected to immunoprecipitation using polyclonal anti-myc or anti-HA antibodies, then immunoblotted using monoclonal anti-HA or anti-myc antibodies, respectively. Cell lysates were also directly immunoblotted using monoclonal anti-HA and anti-myc antibodies to verify similar protein expression levels in experimental and empty expression vector (CMV) control transfections. (A) Co-immunoprecipitation of full-length CHIP and the ARM-Ankyrin-LRR (AAL) portion of LRRK2; (B) Co-immunoprecipitation of full-length CHIP and the ROC-COR-Kinase-WD40 (RCKW) portion of LRRK2; (C) Co-immunoprecipitation of CHIP lacking the U-box domain (CHIPΔU) and the ARM-Ankyrin-LRR (AAL) portion of LRRK2; (D) Co-immunoprecipitation of CHIP lacking the U-box domain (CHIPΔU) and the ROC-COR-Kinase-WD40 (RCKW) portion of LRRK2; (E) Absence of co-immunoprecipitation of CHIP lacking the TPR domain (CHIPΔT) and the ARM-Ankyrin-LRR (AAL) portion of LRRK2; (F) Co-immunoprecipitation of CHIP lacking the TPR domain (CHIPΔT) and the ROC-COR-Kinase-WD40 (RCKW) portion of LRRK2; (G) Geldanamycin impairs the interaction of full-length CHIP with the ARM-Ankyrin-LRR (AAL) portion of LRRK2; (H) Geldanamycin does not disrupt the interaction of full-length CHIP and the ROC-COR-Kinase-WD40 (RCKW) portion of LRRK2. For (G-H), geldanamycin was added into the medium to 1 µM final concentration and incubated for 1 hour prior to cell collection.

Because both CHIP and LRRK2 have multiple domains (see Supplemental Figure S1), we sought to further delineate which domains of CHIP are critical for interacting with different regions of LRRK2. To determine whether full-length CHIP could bind to different regions of LRRK2, we co-transfected HEK293 cells with an expression vector for HA-tagged full-length CHIP (pHA-CHIP) and expression vectors coding for either myc-tagged ARM-ANK-LRR domains of LRRK2 (pMyc-AAL) or myc-tagged ROC-COR-kinase-WD40 domains of LRRK2 (pMyc-RCKW) (see Supplemental Figure S1). Surprisingly, full length CHIP co-immunoprecipitated with both the ARM-ANK-LRR domains of LRRK2 (Figure 2A) and the ROC-COR-kinase-WD40 domains of LRRK2 (Figure 2B).

The primary sequence and crystal structures of CHIP show three domains: an N-terminal tetratricopeptide repeat (TPR) domain, a C-terminal U-box domain and an intervening charged domain [30], [31] (see Supplemental Figure S1). U-box domains are structurally related to RING finger domains, both of which confer E3 ubiquitin ligase activity. Because LRRK2 has previously been shown to bind to the E3 ubiquitin ligase Parkin via its second RING finger domain [32], we hypothesized that the U-box domain of CHIP was responsible for binding to LRRK2. To test whether the U-box domain of CHIP is critical for binding to either half of LRRK2, we co-transfected HEK293 cells with an expression vector for HA-tagged CHIP lacking only the U-box domain (pHA-CHIPΔU) and expression vectors coding for either myc-tagged ARM-ANK-LRR domains of LRRK2 (pMyc-AAL) or myc-tagged ROC-COR-kinase-WD40 domains of LRRK2 (pMyc-RCKW). Unexpectedly, the U-box domain of CHIP is apparently dispensable for binding to either half of LRRK2 because HA-CHIPΔU co-immunoprecipitated with both the ARM-ANK-LRR domains of LRRK2 (Figure 2C) and the ROC-COR-kinase-WD40 domains of LRRK2 (Figure 2D).

The N-terminal TPR domain of CHIP has been shown to specifically bind to several chaperones such as Hsp70 and Hsp90 [33], [34]. Hsp90 has previously been reported to bind to LRRK2 [18], [26], [27] and we have validated this interaction here (Figure 1B, D). This raises the possibility that the CHIP TPR domain binds to LRRK2 indirectly, via Hsp90. To determine whether the TPR domain is necessary for CHIP to interact with either half of LRRK2, we co-transfected HEK293 cells with an expression vector for HA-tagged CHIP lacking only the TPR domain (pHA-CHIPΔT) and expression vectors coding for either myc-tagged ARM-ANK-LRR domains of LRRK2 (pMyc-AAL) or myc-tagged ROC-COR-kinase-WD40 domains of LRRK2 (pMyc-RCKW). Deletion of the CHIP TPR domain completely abolished the interaction between CHIP and the ARM-ANK-LRR domains of LRRK2 based on the absence of co-immunoprecipitation (Figure 2E). By contrast, deletion of the CHIP TPR domain did not disrupt the interaction between CHIP and the ROC-COR-kinase-WD40 domains of LRRK2 because these domains robustly co-immunoprecipitated with CHIPΔT (Figure 2F).

These results, together with our data showing that Hsp90 binds to the ARM domain of LRRK2 (Figure 1B), led us to hypothesize that CHIP binds to the N-terminus of LRRK2 indirectly via Hsp90, which is well-known to bind to the TPR domain of CHIP [35]. Hsp90 is an ATP-dependent molecular chaperone that can be inhibited by compounds such as geldanamycin, which binds to the conserved N-terminal ATP binding pocket and blocks conformational changes required for molecular chaperone function [36]. To test the hypothesis that Hsp90 function is required for CHIP to bind to the N-terminus of LRRK2, we co-transfected HEK293 cells with an expression vector for HA-tagged full-length CHIP (pHA-CHIP) and expression vectors coding for either myc-tagged ARM-ANK-LRR domains of LRRK2 (pMyc-AAL) or myc-tagged ROC-COR-kinase-WD40 domains of LRRK2 (pMyc-RCKW) and incubated the cells with 1 µM geldanamycin for 1 hour prior to cell collection to block Hsp90 function. In contrast to the robust co-immunoprecipitation in the absence of geldanamycin (Figure 2A), full-length CHIP did not co-immunoprecipitate with the myc-tagged ARM-ANK-LRR domains of LRRK2 in the presence of geldanamycin (Figure 2G), indicating that Hsp90 function is required for CHIP to bind to the N-terminal portion of LRRK2. By contrast, geldanamycin did not prevent full-length CHIP binding to myc-tagged ROC-COR-kinase-WD40 domains of LRRK2 (Figure 2H). Together with the data shown in Figure 2F, this suggests that neither the TPR domain of CHIP nor Hsp90 are required for CHIP to bind to the C-terminal portion of LRRK2. Because the U-box domain of CHIP is also dispensable (Figure 2D), the intervening charged domain of CHIP appears to be sufficient for CHIP to interact with the C-terminal portion of LRRK2. This is consistent with our yeast two-hybrid screen results and with the data in Figure 1A, which shows that the intervening charged domain of CHIP binds to the ROC domain of LRRK2.

The above data shows that the TPR domain of CHIP is required for CHIP to bind to the N-terminal portion of LRRK2. To test whether isolated TPR domain is sufficient for binding to LRRK2, we co-transfected HEK293 cells with HA-tagged TPR domain and Myc-tagged N-terminal or C-terminal portions of LRRK2. Co-immunoprecipitation assays showed that isolated CHIP TPR domain binds to the ARM-ANK-LRR domains of LRRK2 but not to the ROC-COR-kinase-WD40 domains of LRRK2 (Supplemental Figure S2A, B). The K30A point mutation within the TPR domain of CHIP that blocks CHIP binding to Hsp90 [37] also blocks binding of the isolated TPR domain to the N-terminus of LRRK2 (Supplemental Figure S2C, D). We further found that isolated CHIP TPR domain binds to full length LRRK2 (Supplemental Figure S2E) and that this was blocked by the K30A point mutation (Supplemental Figure S2F). Consistent with the above data, full length CHIP with the K30A point mutation fails to bind to the ARM-ANK-LRR domains of LRRK2 (Supplemental Figure S2G). As expected, the K30A point mutation does not block binding of full length CHIP to the ROC-COR-kinase-WD40 domains of LRRK2 (Supplemental Figure S2H) or to full length LRRK2 (data not shown). Likewise, geldanamycin does not block binding of full length wild-type CHIP to full length LRRK2 (Supplemental Figure S3).

### CHIP regulates LRRK2 stability

**Figure 3:**
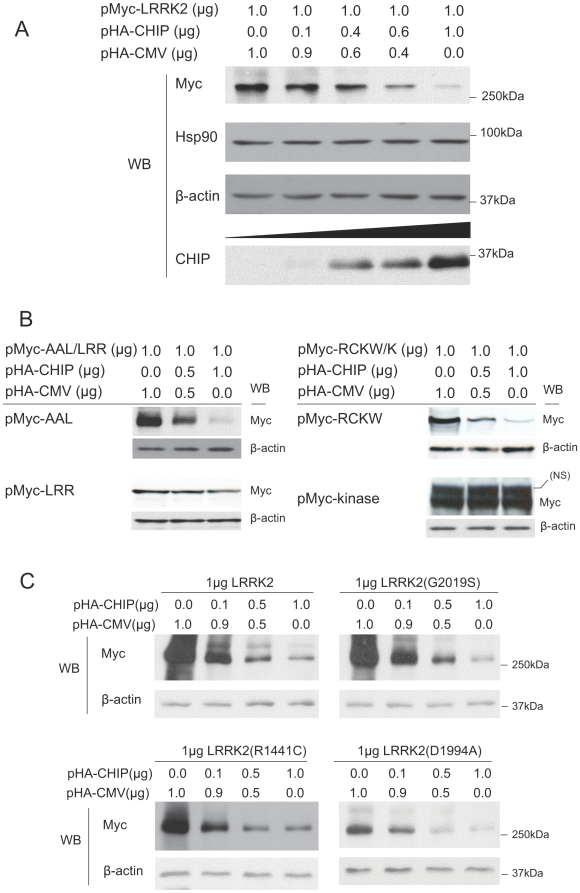
CHIP promotes LRRK2 degradation. (A) CHIP promotes full-length LRRK2 degradation. 1.0 µg of pMyc-LRRK2 was co-transfected with 0, 0.1 0.4, 0.6 or 1.0 µg of pHA-CHIP into 1.5×10^6^ HEK293 cells in 35 mm dishes. Empty vector pHA-CMV was used to normalize the total amount of DNA for each transfection. After 48-hour incubation, equal amounts of cell lysates were used for immunoblotting using anti-myc antibody and the membrane was re-probed using anti-Hsp90, anti-CHIP and anti-β-actin antibodies. (B) Effects of CHIP on the stability of N- and C-terminal portions of LRRK2. 1.0 µg of pMyc-AAL (ARM-Ankyrin-LRR, the N-terminal portion of LRRK2) or pMyc-RCKW (ROC-COR-Kinase-WD40, the C-terminal portion of LRRK2) were co-transfected with 0, 0.5 or 1.0 µg of pHA-CHIP into 1.5×10^6^ HEK293 cells in 35 mm dishes. Empty vector pHA-CMV was used to normalize the total amount of DNA for each transfection. After 48-hour incubation, equal of amounts of cell lysates were used for immunoblotting using anti-myc antibody (the upper panel for each construct). Membranes were stripped and re-probed using anti-β-actin antibody to confirm equal loading. To rule out non-specific down-regulation of the AAL or RCKW portions of LRRK2, isolated leucine rich repeat domain (pMyc-LRR) and kinase domain (pMyc-kinase) of LRRK2 were similarly co-transfected with increasing amounts of CHIP. These domains were not significantly down-regulated by CHIP. NS, non-specific immunoreactive band above the Myc-tagged kinase domain. (C) CHIP destabilizes wild-type and mutant LRRK2. 1 µg of pMyc-LRRK2, LRRK2(G2019S), LRRK2(R1441C) or LRRK2(D1994A) was co-transfected with 0, 0.1, 0.5 or 1.0 µg of pHA-CHIP into 1.5×10^6^ HEK293 cells in 35 mm dishes. Empty vector pHA-CMV was used to normalize the amount of total DNA for each transfection. After 48-hour incubation, the cells were harvested and lysates were immunoblotted with anti-myc and anti-β-actin antibodies to measure the abundance of wild-type and mutant LRRK2.

Because CHIP has been shown to regulate cellular levels of other proteins linked to neurodegenerative disease, such as phospho-tau [38], we investigated the effect of CHIP overexpression on levels of LRRK2. We transiently co-transfected HEK293 cells with a fixed amount of myc-tagged full-length LRRK2 and increasing amounts of full-length CHIP, plus empty vector to normalize all transfections for total DNA. Western analysis of cells harvested 48 hours after transfection showed that overexpression of CHIP decreased LRRK2 levels in a dose-dependent manner (Figure 3A, upper panel). CHIP overexpression had no effect on the levels of endogenous Hsp90 or β-actin (Figure 3A, middle panels). Stripping and re-blotting with an anti-CHIP antibody confirmed the increasing amount of CHIP protein in cells transfected with increasing amounts of CHIP expression vector (Figure 3A, bottom panel). This indicates that CHIP can regulate the stability of LRRK2.

Because we identified CHIP interactions with both N-terminal and C-terminal portions of LRRK2 (Figure 2), we sought to determine whether one or both of these interactions are responsible for destabilization of LRRK2 by CHIP. We transiently co-transfected HEK293 cells with fixed amounts of myc-tagged fragments of LRRK2 and increasing amounts of full-length CHIP, plus empty vector to normalize all transfections for total DNA. Western analysis 48 hours after transfection showed that overexpression of CHIP decreased levels of both myc-tagged ARM-ANK-LRR domains of LRRK2 (pMyc-AAL) and myc-tagged ROC-COR-kinase-WD40 domains of LRRK2 (pMyc-RCKW) in a dose-dependent manner (Figure 3B). To rule out non-specific down-regulation of the AAL or RCKW portions of LRRK2, isolated leucine rich repeat domain (pMyc-LRR) and kinase domain (pMyc-kinase) of LRRK2 were similarly co-transfected with increasing amounts of CHIP. These domains were not significantly down-regulated by CHIP (Figure 3B).

### CHIP destabilizes wild-type and mutant LRRK2

To date, several missense mutations in *LRRK2* have been linked to autosomal dominantly inherited PD (e.g. R1441C/G, Y1699C, G2019S, I2020T), and many additional *LRRK2* variants are being evaluated for conclusive pathogenicity or increased risk for PD [23]. We therefore investigated whether CHIP-mediated degradation of LRRK2 is affected by LRRK2 point mutations that segregate with PD or that ablate the kinase activity of LRRK2. We transfected HEK293 cells with equal amounts of wild-type LRRK2, two PD pathogenic mutants (G2019S, R1441C) or an artificial kinase dead variant of LRRK2 (D1994A) [21] and increasing amounts of CHIP expression vector. Even in the absence of exogenous CHIP (Figure 3C, left lane of each panel), the cellular levels of wild-type and mutant LRRK2 differ considerably in transiently transfected HEK293 cells. The G2019S mutation did not affect LRRK2 levels, but the R1441C mutation diminished LRRK2 levels slightly compared to wild-type. The abundance of the artificial kinase-inactive mutant (D1994A) was significantly decreased relative to wild-type LRRK2, suggesting that the kinase activity of LRRK2 is important for stabilizing LRRK2. In all cases, increasing amounts of CHIP expression vector markedly diminished the abundance of wild-type and mutant LRRK2 relative to β-actin (Figure 3C), indicating that CHIP can destabilize wild-type and mutant LRRK2.

To confirm that none of these LRRK2 mutations disrupt CHIP binding to LRRK2, we conducted co-immunoprecipitation assays using HEK293 cells co-transfected with CHIP and wild-type or mutant LRRK2. Wild-type, D1994A, R1441C and G2019S LRRK2 all co-immunoprecipitated with CHIP (Supplemental Figure S4), indicating that they are able to bind to CHIP either directly or indirectly via common adaptor proteins.

### Both the TPR and U-box domains are essential for CHIP-mediated LRRK2 degradation

**Figure 4:**
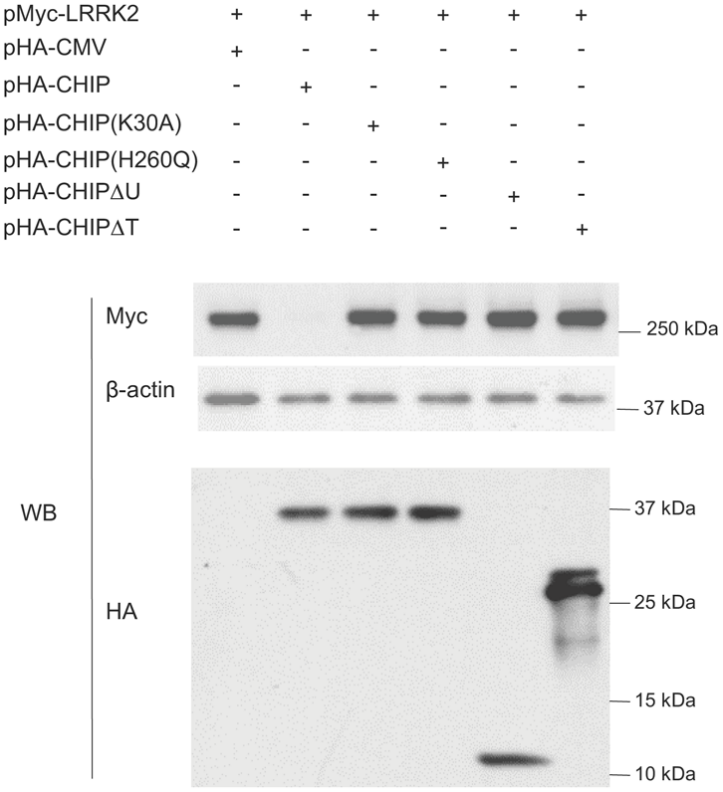
Both TPR and U-box domain are required for LRRK2 degradation. 1 µg of pMyc-LRRK2 and 0.5 µg of pHA-CMV (Lane 1 from left to right) or pHA-CHIP (Lane 2) or pHA-CHIP(K30A) (Lane 3) or pHA-CHIP(H260Q) (Lane 4) or pHA-CHIPΔU (Lane 5) or pHA-CHIPΔT (Lane 6) were co-transfected into 1.5×10^6^ HEK293 cells in 35 mm dishes. 48 hours after transfection, cells were harvested and the lysates were immunoblotted with anti-myc (upper panel), anti-β-actin (middle panel) and anti-HA (lower panel).

Because we found that the N-terminal chaperone interaction (TPR) domain of CHIP and the C-terminal U-box domain of CHIP are not essential for binding to LRRK2 (Figure 2), we examined whether these domains of CHIP are required for CHIP-mediated LRRK2 degradation. To test this, we co-transfected HEK293 cells with Myc-LRRK2 and HA-CHIP or one of the following CHIP mutant constructs: 1) a TPR domain point mutant CHIP(K30A) or TPR domain deletion mutant (CHIPΔT), both of which are unable to interact with Hsp/Hsc70 or Hsp90; and 2) a U-box domain point mutant CHIP(H260Q) or U-box domain deletion mutant (CHIPΔU), both of which are unable to catalyze protein ubiquitin conjugation [37]. Western analysis of cell lysates with anti-myc and anti-HA showed that all the constructs expressed similar amounts of protein (Figure 4). In contrast to wild-type CHIP, which degraded LRRK2, none of the CHIP mutants decreased LRRK2 protein levels (Figure 4). These results indicate that both the chaperone interaction and the ubiquitin ligase activity of CHIP are required for CHIP-mediated degradation of LRRK2 protein.

### CHIP can ubiquitinate LRRK2

**Figure 5:**
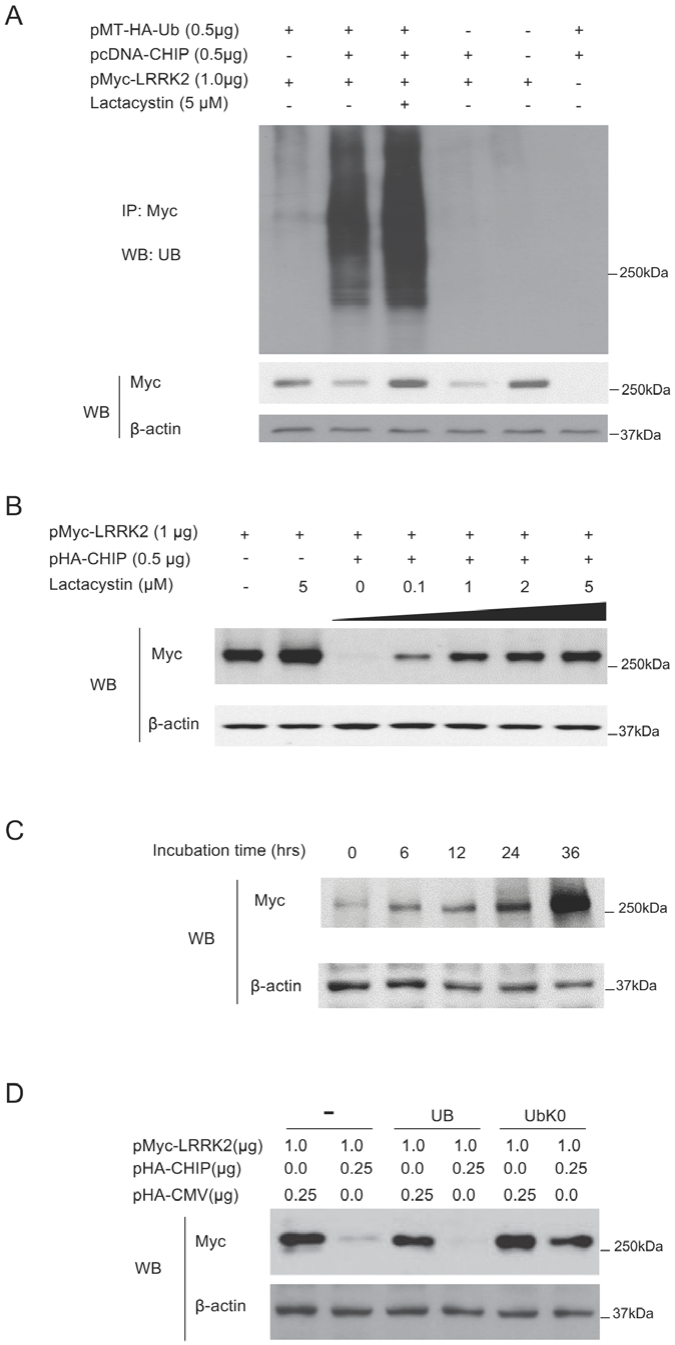
CHIP can ubiquitinate LRRK2 and promote LRRK2 degradation through the ubiquitin proteasome pathway. (A) From Lane 1 to Lane 6 (from left to right), the indicated constructs were co-transfected into 1.5×10^6^ HEK293 cells in 35 mm dishes. In Lane 3, lactacystin was added into the medium to a final concentration of 5 µM immediately following cell transfection. After 24-hour incubation, the cell lysates were immunoprecipitated using a polyclonal anti-myc antibody. The immunoprecipitants were immunoblotted with monoclonal anti-UB antibody (upper panel). The corresponding cell lysates were probed using anti-myc and anti-β-actin (middle and lower panels). (B) 1.0 µg of pMyc-LRRK2 was co-transfected with 0.5 µg pHA-CMV or 0.5 µg of pHA-CHIP into 1.5×10^6^ HEK293 cells in 35 mm dishes. After 24 hours, lactacystin was added to the indicated concentration. DMSO was added in Lane 1 as a negative control. The cells were cultured for another 24 hours. The cell lysates were probed using anti-myc and anti-β-actin. (C) 1.0 pMyc-LRRK2 and 0.5 g of pHA-CHIP were co-transfected into HEK293 cells. After 24 hours, lactacystin was added to the final concentration at 5 µM. The cells were sampled at 0, 6, 12, 24 and 36 hours after addition of lactacystin. The cell lysates were probed using anti-myc and anti-β-actin. (D) HEK293 cells were transfected with 1.0 µg pMyc-LRRK2, with or without 250 ng pHA-CHIP, pMT-HA-Ub, or pCS2-UbK0, as indicated. 48 hours after transfection, LRRK2 protein levels were determined by immunoblotting with anti-myc antibody.

To determine whether CHIP can ubiquitinate LRRK2, mammalian expression vectors for myc-tagged LRRK2, CHIP and HA-tagged ubiquitin (HA-Ub), were co-transfected into HEK293 cells and incubated for 24 hours. Cell lysates were immunoprecipitated with anti-myc antibody and ubiquitinated LRRK2 was detected by immunoblotting with anti-ubiquitin antibody (Figure 5A, upper panel). Cells transfected with all 3 constructs produced typical high-molecular-weight species consistent with polyubiquitinated LRRK2. Incubation of transfected cells with a proteasome inhibitor, lactacystin, caused the accumulation of polyubiquitinated LRRK2 as determined by the increased intensity of the ubiquitin-immunoreactive smear. The transfections lacking LRRK2 or ubiquitin resulted in no signal. Cells transfected with LRRK2 and ubiquitin without CHIP resulted in very weak ubiquitination of LRRK2, perhaps mediated by endogenous CHIP or other ubiquitin ligases. Western analysis of the corresponding cell lysates (without immunoprecipitation) showed that the abundance of LRRK2 was diminished by CHIP and that lactacystin could inhibit this (Figure 5A, middle panel). These results demonstrate the ability of CHIP to ubiquitinate LRRK2.

### CHIP down-regulates LRRK2 levels through the ubiquitin proteasome pathway

To further investigate the extent to which proteasome activity is required for CHIP-mediated LRRK2 degradation, HEK293 cells were co-transfected with pHA-CHIP and pMyc-LRRK2, treated with the proteasome inhibitor lactacystin at concentrations ranging from 0.1 to 5 µM, and immunoblotted to measure the abundance of LRRK2 normalized to actin. As shown in Figure 5B, we observed a lactacystin dose-dependent increase in LRRK2 abundance in cells transfected with both LRRK2 and CHIP. Treatment with 5 µM lactacystin almost completely blocked CHIP-mediated degradation of LRRK2 under conditions in which LRRK2 is almost completely degraded without lactacystin. By contrast, in cells not transfected with CHIP, lactacystin had little effect on LRRK2 abundance. To investigate the kinetics of this effect, we co-transfected HEK293 cells with pMyc-LRRK2 and pHA-CHIP for 24 hours, then added lactacystin to a final concentration of 5 µM and harvested cells 0, 6, 12, 24 and 36 hours after addition of lactacystin. Western analysis showed that up to 36 hours is required for LRRK2 to accumulate in the presence of CHIP and 5 µM lactacystin (Figure 5C). To test whether polyubiquitination is required for CHIP-mediated LRRK2 degradation, we co-transfected HEK293 cells with an artificial ubiquitin variant, UbK0, in which all lysines were replaced by arginines to prevent polyubiquitination [39]. Expression of UbK0, but not wild-type ubiquitin, blocked CHIP-mediated degradation of LRRK2 (Figure 5D), indicating that CHIP stimulates LRRK2 degradation through the ubiquitin proteasome pathway, which requires polyubiquitination.

### Hsp90 overexpression stabilizes LRRK2 and prevents CHIP-mediated LRRK2 degradation

**Figure 6:**
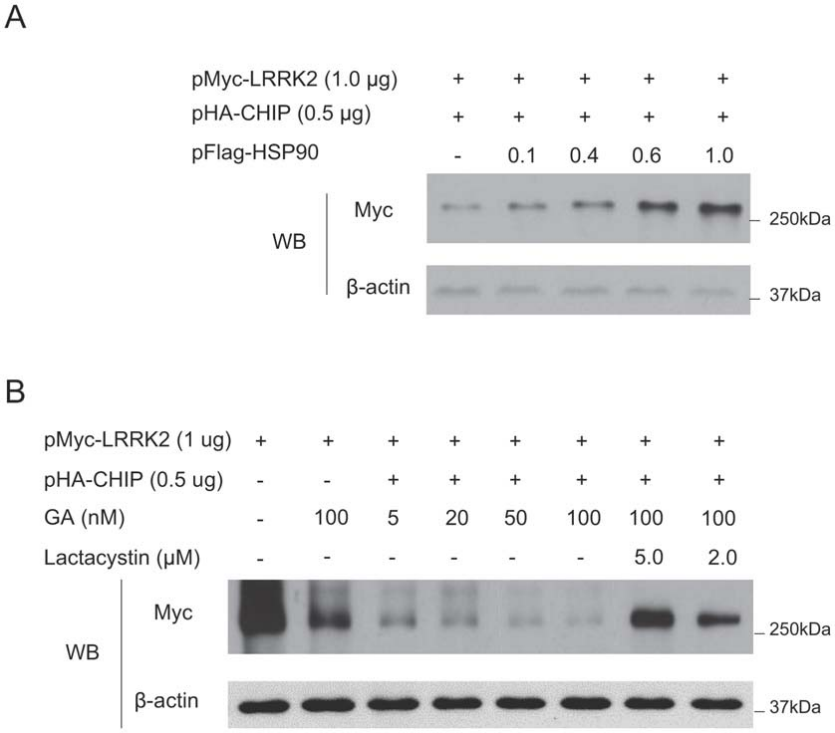
Hsp90 can attenuate CHIP-mediated LRRK2 degradation. (A) 1.0 µg pMyc-LRRK2 and 0.5 µg pHA-CHIP were co-transfected with 0, 0.1, 0.4, 0.6, 1.0 µg pFlag-Hsp90. After 48-hour incubation, the cell lysates were immunoblotted with anti-myc and anti-β-actin antibodies. (B) 1.0 µg of pMyc-LRRK2 was co-transfected with 0.5 pHA-CMV (Lane 1 and 2 from left to right) or 0.5 µg pHA-CHIP (Lane 3–8). 24 hours after cell transfection, GA and lactacystin were added to the indicated concentrations and the cells were cultured for another 24 hours prior to cell collection and western analysis.

It has previously been reported that inhibition of Hsp90 can destabilize mouse LRRK2 [27]. To examine whether Hsp90 overexpression can stabilize LRRK2 even in the presence of CHIP, we co-transfected pMyc-LRRK2, pHA-CHIP and increasing amounts of pFlag-Hsp90 into HEK293 cells. The results indicated that LRRK2 steady-state abundance was increased by overexpressing Hsp90, even in the presence of CHIP (Figure 6A). As expected, inhibition of endogenous Hsp90 with geldanamycin promoted LRRK2 degradation in the presence of CHIP, however, the effect of geldanamycin could be blocked by lactacystin (Figure 6B). Together, these data suggest that Hsp90 prevents CHIP-mediated degradation of LRRK2 via the ubiquitin proteasome pathway.

### LRRK2 forms a protein complex with endogenous CHIP and Hsp90

**Figure 7:**
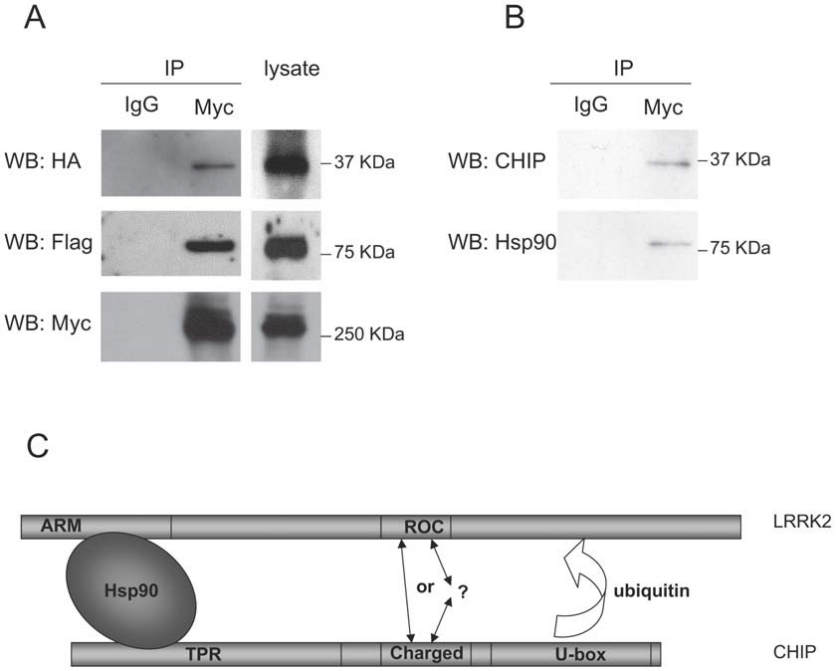
LRRK2, CHIP and Hsp90 form a protein complex. (A). 1 µg of pMyc-LRRK2, pHA-CHIP and pFlag-Hsp90 were co-transfected into 1.5×10^7^ HEK293 cells in 100 mm dishes. Lacatcystin was added at 5 µM to prevent CHIP-mediated degradation of LRRK2. The cells were incubated for 48 hours and the cell lysate was subjected to immunoprecipitatiom using a polyclonal anti-myc antibody or rabbit IgG. The immunoprecipitants and their cell lysates were probed with monoclonal anti-HA, anti-Flag and anti-myc antibodies. (B). 3 µg of pMyc-LRRK2 was transfected into 1.5×10^7^ HEK293 cells in 100 mm dishes. The cells were incubated for 48 hours and the cell lysate was subject to immunoprecipitation using polyclonal anti-myc antibody or rabbit IgG. The immunoprecipitants were probed using anti-CHIP or anti-Hsp90 antibodies. (C) Model of LRRK2 binding to CHIP and Hsp90. CHIP can form a stable complex with LRRK2 by at least two independent protein-protein interactions. The N-terminal TPR domain of CHIP can bind to the N-terminal ARM domain of LRRK2 indirectly via Hsp90. The intermediate charged domain of CHIP can bind to the ROC domain of LRRK2 independent of Hsp90. The U-box domain of CHIP is dispensable for binding to LRRK2 but required for CHIP-mediated ubiquitination and proteasome-dependent degradation of LRRK2.

Given the data presented here, we sought to determine whether LRRK2 forms a protein complex with endogenous CHIP and Hsp90. First, we co-transfected HEK293 cells with pMyc-LRRK2, pFlag-Hsp90 and pHA-CHIP and immunoprecipitated the cell lysates with an anti-myc antibody. As expected, CHIP and Hsp90 were robustly detected by western blotting the immunoprecipitants with anti-HA and anti-Flag antibodies, respectively (Figure 7A). Control immunoprecipitation with IgG verified the absence of non-specific binding. Next, we transfected HEK293 cells with only pMyc-LRRK2, immunoprecipitated the cell lysates with anti-myc antibody and blotted with anti-CHIP and anti-Hsp90 antibodies to detect endogenous CHIP and Hsp90 binding to LRRK2 (Figure 7B). Together, the data show that both overexpressed and endogenous CHIP and Hsp90 can form a protein complex with LRRK2.

### CHIP siRNA decreases endogenous CHIP and increases LRRK2 abundance

Because endogenous CHIP forms a complex with LRRK2, we tested whether decreasing endogenous CHIP by siRNA would affect LRRK2 abundance. We co-transfected HEK293 cells with 50 ng of pMyc-LRRK2 and 0–40 picomoles of CHIP siRNA and then used western blot to measure levels of Myc-tagged LRRK2, endogenous CHIP and β-actin. CHIP siRNA decreased endogenous CHIP and increased LRRK2 abundance in a dose dependent manner (Supplemental Figure S5). Parallel cells transfected with a non-targeting control siRNA showed no effect of control siRNA on CHIP or LRRK2 abundance (Supplemental Figure S5).

## Discussion

Here we report the identification of CHIP as a LRRK2 interacting protein and provide evidence that CHIP can significantly reduce the cellular levels of LRRK2 by ubiquitination and proteasome-dependent degradation. Our data reveal which domains of CHIP and LRRK2 are required or dispensable for this interaction. Surprisingly, we found that CHIP can bind to LRRK2 at least two independent ways. The combined data shown in Figure 1 and Figure 2 indicate that the charged domain of CHIP binds to the ROC domain of LRRK2 and that the TPR domain of CHIP is required for binding to the N-terminal region of LRRK2 that includes the armadillo, ankyrin and leucine-rich repeats. This is the first demonstration that the charged domain of CHIP directly binds to another protein. The TPR domain of CHIP apparently binds to the N-terminal region of LRRK2 indirectly via Hsp90, which is known to bind to the TPR domain of CHIP [35], because blocking Hsp90 function with geldanamycin completely prevented the binding of full-length CHIP to the N-terminal region of LRRK2. By contrast, geldanamycin did not prevent the binding of full-length CHIP to the C-terminal half of LRRK2 (Figure 2), indicating that the interaction between the charged domain of CHIP and the ROC domain of LRRK2 does not require Hsp90 function. We further show that the isolated TPR domain of CHIP is sufficient for binding to the N-terminal half of LRRK2, but not to the C-terminal half of LRRK2 (Supplemental Figure S2). Consistent with the geldanamycin data, the K30A point mutation within the TPR domain that blocks CHIP binding to Hsp90, also blocks binding to the N-terminal half of LRRK2 (Supplemental Figure S2).

We propose a model in which the charged domain of CHIP binds to the ROC domain of LRRK2 either directly or indirectly, but independent of Hsp90, while the TPR domain of CHIP binds to the N-terminus of LRRK2 indirectly, via Hsp90 (Figure 7C). Surprisingly, the U-box domain of CHIP, which is structurally related to RING finger ubiquitin ligase domains, is apparently dispensable for CHIP binding to LRRK2 (Figure 2C, D). This mode of binding differs from the previous finding that LRRK2 binds to the second RING finger domain of the E3 ubiquitin ligase Parkin [32]. LRRK2 reportedly enhances the auto-ubiquitination activity of Parkin, however, Parkin does not ubiquitinate LRRK2 [32]. By contrast, our data indicates that CHIP ubiquitinates LRRK2 (Figure 5). Because CHIP has also been reported to form a complex with Parkin and to enhance the ubiquitin ligase activity of Parkin [40], it is possible that the reported interaction between LRRK2 and Parkin is mediated by CHIP.

Our most striking finding is that CHIP decreased the stability of LRRK2 in a dose-dependent manner (Figure 3). Because we found that CHIP can bind directly to LRRK2 and cause the ubiquitination and proteasome-dependent degradation of LRRK2, this is a likely mechanism by which CHIP destabilizes LRRK2. CHIP-mediated degradation of LRRK2 appears to require the chaperone interaction function of CHIP because we did not observe any LRRK2 destabilization by a TPR domain point mutant of CHIP (K30A) or by a TPR domain deletion mutant of CHIP (CHIPΔT), both of which are unable to interact with Hsp/Hsc70 or Hsp90 [37]. CHIP-mediated degradation of LRRK2 appears to also require the ubiquitin ligase activity of CHIP because we did not observe any LRRK2 destabilization by a U-box domain point mutant of CHIP (H260Q) or by a U-box domain deletion mutant of CHIP (CHIPΔU), both of which are unable to catalyze protein ubiquitin conjugation [37].

Our studies showed similar CHIP-mediated degradation of wild-type LRRK2 and LRRK2 with disease-linked point mutations (Figure 3C), therefore the pathogenic mechanisms of the mutations we tested do not likely involve diminished CHIP-mediated degradation. These findings are consistent with previous studies that found no effect of LRRK2 mutations on ubiquitination, proteasomal degradation, steady-state levels or turnover of LRRK2 [19], [27]. However, we found that the kinase activity of LRRK2 is important for stabilizing cellular levels of LRRK2 because an artificial variant of LRRK2 lacking kinase activity was markedly and consistently destabilized even in the absence of exogenous CHIP (Figure 3C). The diminished abundance of R1441C and D1994A LRRK2 in the absence of exogenous CHIP could be due to degradation mediated by endogenous CHIP, other degradation mechanisms or to other possible effects such as altered expression. LRRK2 kinase activity does not appear to be required for CHIP mediated-degradation because CHIP decreased the abundance of the artificial kinase-inactive mutant (D1994A) LRRK2 in a dose-dependent manner (Figure 3C), although this experiment cannot rule out the possibility that CHIP affects expression rather than degradation.

CHIP has previously been shown to promote the proteasomal degradation of other proteins implicated in neurodegenerative diseases such as tau, Aβ, α-synuclein oligomers and proteins with expanded polyglutamine repeats [34], [41], [42], [43], [44], [45], [46], [47], [48]. During revision of this manuscript, Ko *et al.* reported that CHIP regulates LRRK2 ubiquitination, degradation and toxicity [49]. Similar to our findings, Ko *et al.* found that CHIP binds to both wild-type and mutant LRRK2 and promotes the ubiquitination and proteasomal degradation of LRRK2. Our findings differ in that we identified two independent means of CHIP binding to LRRK2: an indirect interaction between the TPR domain of CHIP and the ARM domain of LRRK2, likely via Hsp90, and an interaction between the charged domain of CHIP and the ROC domain of LRRK2, which is either direct or indirect via a common adaptor protein. The ARM domain interaction may not have been detected by Ko *et al.* because the isolated full length ARM domain was not shown to be tested for CHIP binding.

In addition to CHIP, our yeast two-hybrid analysis also identified Hsp90 as a LRRK2 binding protein. Several previous studies have shown that Hsp90 can bind to full length LRRK2 or to the kinase domain of LRRK2 [18], [26], [27]. Here we show for the first time that Hsp90 can also bind to the ARM domain at the N-terminus of LRRK2. ARM domains mediate protein-protein interactions and are found in proteins with diverse functions including cytoskeletal regulation and intracellular signaling [50]. Here we propose a model in which the ARM domain stabilizes LRRK2 in part by binding to Hsp90 (Figure 7C). Because the TPR domain of CHIP is well-known to bind to Hsp90 [35], it is likely that a portion of the LRRK2-Hsp90 complex will also be associated with CHIP. LRRK2 apparently forms a stable complex with Hsp90 and CHIP because we successfully immunoprecipitated endogenous CHIP and endogenous Hsp90 together with LRRK2 (Figure 7).

Our data (Figure 6) confirm that overexpression of Hsp90 stabilizes LRRK2 and that inhibition of endogenous Hsp90 by geldanamycin destabilizes LRRK2, as previously reported [27]. Importantly, we show for the first time that Hsp90 overexpression impairs CHIP-mediated degradation of LRRK2 (Figure 6A) and that inhibition of endogenous Hsp90 by geldanamycin enhances CHIP-mediated degradation of LRRK2 (Figure 6B). According to our model (Figure 7C), inhibition of endogenous Hsp90 by geldanamycin prevents CHIP binding to the N-terminal half of LRRK2 (Figure 2G) but does not inhibit the Hsp90-independent binding of CHIP to the C-terminal half of LRRK2 (including the ROC domain), which promotes the ubiquitination and proteasome-dependent degradation of LRRK2. In the presence of excess CHIP, Hsp90-dependent binding of CHIP to the ARM domain of LRRK2 can also destabilize LRRK2. We hypothesize that the stability of LRRK2 depends on the ratio of the cellular abundance and binding availability of Hsp90 and CHIP.

The fact that LRRK2 mutations cause both familial and apparently sporadic forms of PD with typical clinical symptoms and late age-at-onset [51] highlights the significance of age as a causative factor for both familial and idiopathic PD. An important question is whether the cellular abundance and binding availability of Hsp90 and CHIP change with age and whether this contributes to PD risk, perhaps by increasing LRRK2 abundance, aggregation or neurotoxicity. It has been proposed that the ability of CHIP to degrade potentially neurotoxic misfolded, damaged or mutated proteins might diminish with age [25]. Our findings raise the possibility that diminished CHIP-mediated degradation of LRRK2 in aged or stressed neurons may contribute to sporadic PD as well as familial PD in patients bearing LRRK2 mutations. Further studies of the detailed mechanisms that control LRRK2 levels may lead to the development of PD therapies that exploit these mechanisms to degrade wild-type or mutant LRRK2 and thereby mitigate neurotoxicity.

## Supporting Information

Figure S1 Diagrams of LRRK2 and CHIP constructs used for experimental analyses. The domains of LRRK2 and CHIP are shown as labeled boxes. The locations of all the individual amino acid point mutations or variants used for analyses are shown in full length LRRK2 and full length CHIP, although each construct used for experimental analysis was either wild-type or mutated at a single site. Myc epitope tags were used for all constructs expressing full length or partial fragments of LRRK2. HA epitope tags were used for all constructs expressing full length or partial fragments of CHIP. Abreviations: ARM, armadillo repeat domain of LRRK2; ANK, ankyrin repeat domain of LRRK2; LRR, leucine-rich repeat domain of LRRK2; ROC, Ras of complex domain of LRRK2; COR, C-terminal of Roc domain of LRRK2; Kinase, kinase domain of LRRK2; WD40, WD40 domain of LRRK2; TPR, tetratricopeptide repeat domain of CHIP; CD, charged domain of CHIP; U-box, U-box domain of CHIP.
(0.37 MB EPS)

Figure S2 The TPR domain of CHIP is sufficient to bind to the N-terminal portion of LRRK2 and the K30A point mutation that disrupts CHIP binding to Hsp90 disrupts CHIP binding to the N-terminal portion of LRRK2 but not to the C-terminal portion of LRRK2. The indicated constructs (0.5 µg for each) were co-transfected into 4.5×10^6^ HEK293 cells in 60 mm dishes. HEK293 cell lysates were subjected to immunoprecipitation using polyclonal anti-myc or anti-HA antibodies, then immunoblotted using monoclonal anti-HA or anti-myc antibodies, respectively. Cell lysates were also directly immunoblotted using monoclonal anti-HA and anti-myc antibodies to verify similar protein expression levels in experimental and empty expression vector (CMV) control transfections. (A) Co-immunoprecipitation of isolated CHIP TPR domain and the ARM-Ankyrin-LRR (AAL) portion of LRRK2; (B) Absence of co-immunoprecipitation of isolated CHIP TPR domain and the ROC-COR-Kinase-WD40 (RCKW) portion of LRRK2; (C) Absence of co-immunoprecipitation of K30A point mutant CHIP TPR domain and the ARM-Ankyrin-LRR (AAL) portion of LRRK2; (D) Absence of co-immunoprecipitation of K30A point mutant CHIP TPR domain and the ROC-COR-Kinase-WD40 (RCKW) portion of LRRK2; (E) Co-immunoprecipitation of isolated CHIP TPR domain and full length LRRK2; (F) Absence of co-immunoprecipitation of K30A point mutant full length CHIP and full length LRRK2; (G) Absence of co-immunoprecipitation of K30A point mutant full length CHIP and the ARM-Ankyrin-LRR (AAL) portion of LRRK2; (H) Co-immunoprecipitation of K30A point mutant full length CHIP and the ROC-COR-Kinase-WD40 (RCKW) portion of LRRK2.
(2.31 MB EPS)

Figure S3 Geldanamycin does not impair the association of full length CHIP with full length LRRK2. The indicated constructs (0.5 µg for each) were co-transfected into 4.5×10^6^ HEK293 cells in 60 mm dishes. Geldanamycin was added into the medium to 1 µM final concentration and incubated for 1 hour prior to cell collection. HEK293 cell lysates were subjected to immunoprecipitation using polyclonal anti-myc or anti-HA antibodies, then immunoblotted using monoclonal anti-HA or anti-myc antibodies, respectively. Cell lysates were also directly immunoblotted using monoclonal anti-HA and anti-myc antibodies to verify similar protein expression levels in experimental and empty expression vector (CMV) control transfections.
(0.49 MB EPS)

Figure S4 CHIP binds to wild-type, G2019S, R1441C and D1994A LRRK2. The indicated constructs (0.5 µg for each) were co-transfected into 4.5×10^6^ HEK293 cells in 60 mm dishes. HEK293 cell lysates were subjected to immunoprecipitation using polyclonal anti-myc or anti-HA antibodies, then immunoblotted using monoclonal anti-HA or anti-myc antibodies, respectively. Cell lysates were also directly immunoblotted using monoclonal anti-HA and anti-myc antibodies to verify similar protein expression levels in experimental and empty expression vector (CMV) control transfections. (A) Co-immunoprecipitation of full-length CHIP and full length wild-type LRRK2; (B) Co-immunoprecipitation of CHIP and G2019S mutant LRRK2. (C) Co-immunoprecipitation of CHIP and R1441C mutant LRRK2. (D) Co-immunoprecipitation of CHIP and D1994A variant LRRK2.
(1.00 MB EPS)

Figure S5 CHIP siRNA stabilizes LRRK2. (A). 0.05 µg of pMyc-LRRK2 was co-transfected with 0-40 picomoles of CHIP siRNA into 1.5×10^5^ neuroblastoma SH-SY5Y cells in 24-well format. After 48-hour incubation, the cells were harvested and the proteins blotted with antibodies to Myc, CHIP and β-actin. (B). Parallel cells were similarly co-transfected using 0.05 µg of pMyc-LRRK2 and 0–40 picomoles of non-targeting siRNA in place of CHIP siRNA and western blotted to confirm no effects on the levels of endogenous CHIP or transfected Myc-LRRK2.
(0.71 MB EPS)
